# Association between Physical Activity and Mood States of Children and Adolescents in Social Isolation during the COVID-19 Epidemic

**DOI:** 10.3390/ijerph17207666

**Published:** 2020-10-21

**Authors:** Xinxin Zhang, Wenfei Zhu, Sifan Kang, Longkun Qiu, Zijun Lu, Yuliang Sun

**Affiliations:** Department of Exercise Science, School of Physical Education, Shaanxi Normal University, Xi’an 710119, China; zhangxx9606@snnu.edu.cn (X.Z.); wzhu@snnu.edu.cn (W.Z.); sifank0405@snnu.edu.cn (S.K.); qlk19971027@snnu.edu.cn (L.Q.); luzijun@snnu.edu.cn (Z.L.)

**Keywords:** COVID-19, social isolation, physical activity, mental health

## Abstract

The COVID-19 epidemic adversely affects the lifestyle of people. This study aimed to examine the impacts of social isolation on physical activity (PA) levels and mood states of children and adolescents and to explore the correlation between them during the COVID-19 epidemic. A total of 9979 children and adolescents (11.63 ± 1.23 years old) from Yan’an, China volunteered to participate in this study and completed online questionnaires. PA and mood states were measured by International Physical Activity Questionnaire Short Form (IPAQ-SF) and Profile of Mood States (POMS), respectively. The results showed that the mean of students’ moderate-to-vigorous PA (MVPA) was 23.19 min per day. The total mood disturbance in the moderate and high-level PA groups were significantly lower than those in the low-level PA group (*p* < 0.05). Additionally, boys and girls had significant differences in PA levels (*p* < 0.01), and the PA levels of students in different grades were also significantly different (*p* < 0.01). Meanwhile, boys’ mood states were worse than girls’. The Grade 4 in primary schools had the worst mood states while Grade 5 had the best mood states. The results suggested that the MVPA of students has dropped badly, compared with the results of previous studies investigated in normal times. In addition, the PA level had a significantly positive impact on the mood states of children and adolescents during the COVID-19 epidemic. Sex and grades were factors which affected the PA levels and mood states. This study can help policymakers and healthcare professionals understand PA and mood states of Chinese children and adolescents during the epidemic. We should pay attention to the changes in PA levels and mood states of children and adolescents.

## 1. Introduction

The novel coronavirus disease (COVID-19) outbreak began in 2019. In just a few months, most countries were severely affected [1]. On 30 January 2020, the World Health Organization (WHO) officially declared COVID-19 incident as an international public health emergency [2]. More than 200 countries and regions around the world were affected by COVID-19, with a total of 28,871,176 confirmed patients and a total of more than 921,801 deaths by 15 September 2020. It has a huge negative impact on the global society, economy and life, which also seriously threatens people’s lives. In response to this epidemic, governments in many countries, especially China, have implemented “partial blockade” and “social isolation” strategies by closing schools, factories and other public places. People are required to stay at home. The strategies reduced cross-infection effectively. However, a long period of social isolation has a negative influence on individuals’ lives.

Nationwide school closures have been one of the most important policy interventions adopted by the Chinese government. Due to a long duration of social isolation, repeated delays in the start of school, and remote online learning in front of screens, there existed a great threat to the daily routines, as well as the physical and mental health of children and adolescents. Although social isolation and confinement are effective measures to deal with the spread of the new coronavirus, however, the ongoing lockdown across the country was bound to change the way of life of general people [3]. Recent studies reported that the social isolation seriously affected people’s lifestyles, such as PA levels, eating habits, alcohol consumption, mental health, quality of sleep and so on [4,5,6,7,8]. Thus, social isolation during the COVID-19 epidemic had a huge impact on public health of children and adolescents [9,10]. Previous research has shown that, even under normal life circumstances, physical activity (PA) for children and adolescents is far from meeting the standard recommended by WHO [11,12,13], which will cause health problems, such as overweight and obesity [14]. During the COVID-19 epidemic, the situation might be exacerbated, because being directly exposed to nature (“green exercise”) is an important reason for the increase in PA levels [15] while the home isolation leads to decrease in children and adolescents’ PA levels [16].

Emotional problem is another prominent social concern. Previous studies have shown that a long period of social isolation has a great negative psychological impact on residents [17], causing negative moods such as depression, stress, and anxiety [18]. Several recent studies have conducted investigations on psychological problems in the context of COVID-19 [19,20]. It was found that more than 35% of people showed psychological distress [21], and 53.8% people showed negative emotional influence during the COVID-19 epidemic [22]. Although the psychological problems due to the COVID-19 epidemic have been studied, limited research has been conducted for children and adolescents. The increase in the spread of COVID-19, was bound to bring a great mood burden to children and adolescents, which will adversely affect their physical and mental health development. Therefore, research on the mood of children and adolescents in the context of COVID-19 has become particularly important.

PA can promote positive mood [23], help maintain a healthy weight, and establish the self-esteem of children and adolescents [24]. Vigorous-intensity PA can significantly reduce adolescents’ negative moods such as stress, anxiety, and depression [25]. To the contrary, excessive PA can also be harmful to mental health [23]. Given a long-term isolated environment, with reductions in daily movement and huge academic learning pressure, it is very necessary to investigate the association between PA levels and emotional state of students during the epidemic to promote the healthy growth of children and adolescents. To date, most current studies on the relationship between PA and mood states were focused on adolescents [26], and few studies on children have been found.

This study aimed to examine the impacts of social isolation on PA levels and mood states of children and adolescents and to explore the correlation between them during the COVID-19 epidemic. We hypothesized that the students’ PA levels dropped, and PA was associated with the mood states among Chinese children and adolescents during the COVID pandemic, and higher PA was related with low levels of mood disturbances. The finding will help develop methods and strategies to support people to overcome the outbreak of pandemics or social isolation and provides advice for policymakers and health care professionals to enhance the practice of PA in future public health emergencies.

## 2. Materials and Methods

### 2.1. Sample and Study Design

This study was a cross-sectional study conducted in the period between 8 and 15 March, after the Director-General of WHO declared the COVID-19 outbreak a public health emergency. A one-week online questionnaire survey was conducted on the Wenjuanxing questionnaire platform (https://www.wjx.cn/), a widely used system for posting online questionnaires and collecting data in China. To ensure the validity and reliability, all children and adolescents were required to fill the questionnaires under the guidance of their parents.

All the participants were the students from Grade 4 to 6 of primary schools in Yan’an City, Shaanxi province, China. It is a major agricultural region. In this area, children and adolescent have nearly 160 min PA per week in the obligatory physical education (PE) classes in “Normal” time. Additionally, most kids and youths participate in different forms of sport or recreation out of school. Besides popular games worldwide like soccer and basketball, some local traditional folk sports are well organized during the students’ leisure time or in the summer/winter vocation, such as Ansai waist-drum, Luohe war-drum, Yangko dance and so on. However, during the COVID-19 epidemic, the students had to stay at home and study online. The PE classes existed in name only.

A total of 9979 students, with an average age of 11.63 ± 1.23 years, participated in this study. The age of students was calculated via the following equation:

Age = (Date of survey − Date of birth) ÷ 365.25

All participants in this study signed informed consent, and the study has been approved by the Shaanxi Normal University Ethics Committee (202016001). The questionnaires included three domains: sociodemographic information (age, sex, grade, living area, daily routine, etc.), PA, and mood states. Among the 9979 valid questionnaires, there were 5131 from boys and 4848 from girls, accounting for 51% and 49%, respectively. The sample was composed of 3764 participants from Grade 4 (37.72%), 3126 from Grade 5 (31.33%) and 3089 from Grade 6 (30.95%). Among the participants, most were between 10 and 13 years old (Table 1).

### 2.2. Measures

Due to the special circumstances during the COVID-19 epidemic, normal daily PA of students in school could not be carried out. Most PA questionnaires for students did not apply to the evaluation of home-based PA levels during social isolation. We chose the International Physical Activity Questionnaire Short Form (IPAQ-SF) to investigate the PA level of children and adolescents. The reliability and validity of IPAQ-SF have been verified under different backgrounds in different countries [27], and it has been used to estimate the PA level of adolescents [28]. A recent study has used the IPAQ-SF to estimate the indoor PA levels in physiotherapy professionals and students during the COVID-19 pandemic [29]. The IPAQ-SF required the participants to recall the number of days they performed each activity (frequency) and the length of time (duration) they were involved in each daily activity in the last 7 days, as well as the average time spent on sedentary behaviors. The results were used to estimate the amount of PA per week, expressed in Metabolic Equivalent Task minutes per week (MET-min/week), and the MET assignments for each intensity of PA were: walking (3.3 METs), moderate (4 METs), and vigorous (8 METs). According to the official IPAQ–SF scoring protocol, participants were classified into three levels according to the cut-off of total metabolic equivalent task weekly: low, moderate, and high [27].

The Profile of Mood States (POMS) revised by Beili Zhu was used to conduct mood tests on the participants, and its reliability and validity have been confirmed [30,31]. The scale contained a total of seven subscales, and participants were indicated to “describe how they have been feeling during the past week, including today” on 5-point scales (anchors: 0 = not at all; 1 = a bit less; 2 = intermediate; 3 = more; 4 = extremely) [32]. From these subscales, the total mood disorder (TMD) score was calculated by subtracting the positive mood scale (vigor and self-esteem) from the negative mood scale (tension, depression, anger, fatigue, and confusion). Scores can range from 0 to 200, with higher scores indicating a higher degree of mood disturbance.

### 2.3. Statistical Analysis

All analyses were performed using SPSS software (version 23.0). PA and POMS variables were presented as mean ± standard deviation (SD). As PA variables were non-normally distributed, non-parametric tests (Mann–Whitney U test and Kruskal–Wallis test) were used to compare the PA differences between sex and grade group. Comparison of mood states of different sexes was calculated by independent sample *t*-test. One-way ANOVA (post-hoc test: Turkey) was used to compare the mood states in different grade groups. General linear regression was used to examine the association between PA-level and mood states, controlling for sex and grade. The statistical significance was set at *p* < 0.05 (two-tailed).

## 3. Results

The Quartile 1, median, and Quartile 3 of total PA were 0, 560.0, and 1800.0 MET-min/week, respectively. The analysis presented in Table 2 was the moderate-to-vigorous physical activity (MVPA) of the students was 1193.02 ± 1621.88 MET-min/week, which was 23.19 ± 33.52 min per day in MVPA. Vigorous PA accounted for the largest proportion (42.74%). Walking PA accounted for 24.19%, which was the lowest. The MVPA for boys was 1186.62 ± 1670.81 MET-min/week. Among them, vigorous PA accounted for 44.40%, which was the largest. Walking PA accounted for the lowest (23.93%). The total metabolic equivalent of PA for girls was 1199.79 ± 1568.58 MET-min/week. Among them, vigorous PA accounted for 41.08%, accounting for the highest, walking PA accounted for the lowest, 24.46%. Statistically significant differences of MVPA and moderate PA existed between boys and girls (*p* < 0.01), and boys had less MVPA and moderate PA than girls.

It was found that boys had significantly higher TMD scores than girls (*p* < 0.01). The four negative subscales of POMS (Anger, Fatigue, Depression, and Confusion) in boys were significantly higher than those of girls (*p* < 0.01). No significance was found in the positive subscales between boys and girls (*p* > 0.05).

The analysis presented in Table 3 shows that there were statistically significant differences in PA levels among students of different grades (*p* < 0.01). Grade 4 had the highest TMD score and Grade 5 had the lowest TMD score. In the negative mood subscale, the Anger subscale score in Grade 4 was significantly higher than that in Grade 5 (*p* < 0.05); the total score of Fatigue subscale in Grade 6 was significantly higher than that in Grade 5 (*p* < 0.01); the total scores of the Depression subscales in Grades 4 and 6 were significantly higher than those in Grade 5; the Confusion subscale score was highest in Grade 4 and lowest in Grade 6. On the positive mood subscale, the Vigor subscale scored highest in Grade 6 and lowest in Grade 4, but the Self-esteem subscale scored significantly higher in Grade 4.

The analysis presented in Table 4 was that a significant relationship existed between PA and mood states in children and adolescents. In the negative mood subscale, the total scores of Depression, Confusion, Anger, and Fatigue in the moderate and high-level PA groups were significantly lower than those in the low-level PA group (*p* < 0.01), respectively. In the positive mood subscale, the total scores of Vigor and Self-esteem of the moderate and high-level PA group were significantly higher than those of the low-level PA group (*p* < 0.01), respectively. The study also found that there was no interaction between different sexes, grades, and PA levels (*p* > 0.05).

## 4. Discussion

This study was one of the first to reveal the association between PA and mood states of children and adolescents of different sexes and grades in the context of the COVID-19 outbreak. Our study found the MVPA of students has considerably declined, compared with the results of previous studies [33,34,35,36], which will be discussed in detail in the following parts. Furthermore, we also found that PA was related to the mood states of children and adolescents, and lower PA levels showed higher scores in negative mood states. During the epidemic, boys showed lower PA and emotional state than girls. However, among different grades, the PA and mood states has been shown a mixed relationship. Students in Grade 4 had the lowest PA level and the worst mood states, while those in Grade 5 had the best mood states with a moderate PA level. Although strict social isolation can effectively control the outbreak, it can adversely affect both physical and mental health. The COVID-19 pandemic provided an opportunity to redesign the study and evaluate the impact of isolation measures (including the transition to online learning) on mood states and PA patterns. Our study reveals the relationship between PA level and mood states of children and adolescents in the social isolation period in the COVID-19 epidemic, and provides theoretical guidance for related health promotion and psychological rehabilitation of children and adolescents in the COVID-19 epidemic or post-epidemic era.

Recent study has found that although it is generally recommended to maintain physical exercise during the pandemic, social isolation have greatly reduced the PA level of male and female students [33]. Even during non-epidemic periods, social isolation also had a huge impact on PA. [34]. Our study results showed that children and adolescents’ PA was at a very low level during the COVID-19 epidemic. A recent non-epidemic period study investigated the data of the International Children’s Accelerometer Database (ICAD) 2.0 and found that the children spent about 35 min per day engaging in MVPA [35]. Another study in China found that children and adolescents spent 41.11 min per day on MVPA [36]. However, our study found that children and adolescents spend only 23.19 min per day on MVPA during the epidemic. This indicated that the PA level of children and adolescents has obviously gone down due to the epidemic. This was consistent with previous study results [37,38]. A long period of social isolation is bound to cause a sudden change in living habits and change people’s way of life [39]. In the current study, the participants were all students in Grade 4–6 in primary schools. During the COVID-19 epidemic, schools organized online classes to study, and students’ daily activities were reduced (such as walking between classes and campus) [40]. At the same time, there were statistically significant differences in PA levels among children and adolescents of different sexes and grades, indicating the public health restrictions would potentially influence PA for different populations [41]. During the epidemic, the MVPA of girls was significantly higher than that of boys. The reason for this phenomenon was likely to be the different characteristics of boys and girls. Under the influence of spirit and sense of competition [38,42], most boys are more fond of taking part in sports activities with higher intensity and competition [43,44], such as football, basketball, or running outside. However, due to strict restrictions during the epidemic, boys can only perform indoor exercises at home, which greatly affects the boys’ PA level [40]. Total MVPA of girls during the COVID-19 epidemic may also be related to fundamental changes in daily schedules and habits [38]. Most girls are more inclined to do some workouts at home, such as aerobics, dancing, yoga and so on. Meanwhile, in China, girls were more likely to undertake some housework at home [45], thus they may have more moderate and light PA than boys [46]. In previous studies, most studies have found that boys have higher levels of PA than girls during non-COVID-19 epidemic periods [47,48]. That was contrary to our research results, but it also reflected the significant impact of the COVID-19 epidemic on the PA among children and adolescents. Considering various diseases that may be caused by insufficient PA [37,49,50,51], relevant departments should combine relevant measures to further explore the specific influencing factors. A previous study has shown that the level of PA has dropped significantly during the transition from elementary to middle school [52]. In our study, statistically significant differences were also found in the PA levels of children and adolescents in different grades; however, students from Grade 6, who are going to transit from primary to middle school, had the highest total MVPA. The reason for the inconsistent results might be that the social isolation overshadowed the differences of the PA levels of students in different grades to some extent. However, other factors may affect more aspects, such as different lessons for different grades [53], sex, age and breakfast intake [54] and other family environment factors. Further discussion on specific reasons should be carried out based on the actual situation in the future.

In our study, compared with girls, boys showed higher TMD score and worse mood states. It is worth noting that this finding was contrary to previous studies. Previous studies have suggested that during the COVID-19 epidemic, girls were more affected psychologically and their negative mood was higher [55,56]. Epidemiological studies have found that women are more likely than men to suffer from depression and other negative mood disorders [57,58]. Furthermore, one study in China has found no statistically significant differences between sexes [59]. The reason why our results were different from those of previous studies may be related to the age of the participants. Most of the participants in previous studies were adults, and there were relatively fewer studies on children and adolescents. The social isolation strategy during the COVID-19 epidemic was bound to reduce the outdoor recreation time of children and adolescents. Boys had a greater resistance to social isolation than that of girls, which had a greater impact on mood states. For different grades in primary schools, our study found that during the COVID-19 epidemic period, Grade 4 had the worst mood states and Grade 5 had the best mood states. The results of our study may be due to a number of factors. During social isolation, children and adolescents were unable to carry out normal study and life, and online learning was adopted for course learning. A long duration of screen learning may have had a negative impact on mental health [26,60], resulting in a negative mood. Meanwhile, factors such as health status, psychological interventions [22] and rumors [61] during the COVID-19 epidemic may be important reasons affecting the mental health of children and adolescents. Besides, in our study, it was found that children and adolescents had lower TMD scores and their mood states were good during the COVID-19 epidemic. This may be because during the period of our investigation, the epidemic situation in the investigation area has been effectively controlled, and most children and adolescents have adapted to living in isolation at home.

Our analysis found that the PA was significantly associated with the mood states of children and adolescents during the COVID-19 epidemic. The higher the level of PA, the better the mood states. This was consistent with some previous studies during non-epidemic periods [26,62]. There were also previous studies reported PA or physical exercise had little effect on mood states [63,64,65,66,67,68]. However, most of these previous studies were aimed at specific mental health problems (such as depression, etc.) [64,65,66], or psychological mechanisms [69]. In this study, we mainly focused on the relationship between PA and mood states of a large sample of children and adolescents. This may be an important reason for the difference in results. Besides, previous studies have found that many potential factors can influence the relationship between PA and mood states, such as motion perception [70], social background [71], flexibility [72], etc. Therefore, the causes of the difference in results cannot be generalized, and a comprehensive discussion should be conducted by combining various factors. In the meantime, our study found no interaction between sex, grade and PA level. In the future, we should conduct intervention studies on various influencing factors to explore the differences in the effects of different levels of PA on mood states [26]. Relevant studies under special background are expected to provide effective reference for relevant health promotion and policy implementation.

The strengths of our study included: first, this was one of the first studies that investigated the relationship between PA and mood states in children and adolescents during the COVID-19 epidemic. Second, the data collection sample size was large and the response rate was high, which can provide references for relevant health promotion and policy implementation. Third, the questionnaire was filled out under the guidance of parents, which had high reliability and validity. The current study also had some limitations. First, this study was a cross-sectional survey, and we were unable to determine the causal relationship between PA and mood factors. Secondly, during the COVID-19 epidemic, the physical activity in our participants was limited and their lifestyle was sedentary. The results of physical activity were not normally distributed, and the data were skewed to the lower ends. Therefore, non-parametric tests were used in our study. Third, the IPAQ-SF questionnaire we used was not completely suitable for the assessment of the PA of children and adolescents. However, during the COVID-19 epidemic, most of the questionnaires did not meet the current research status since in-person physical education classes had been cancelled. After comprehensive consideration, the use of the IPAQ-SF questionnaire can also reflect the PA levels of children and adolescents to a certain extent. In the future, appropriate questionnaires should be developed for PA surveys in special periods and home environments to conduct effective surveys.

## 5. Conclusions

Due to the social isolation in the COVID-19 epidemic, children and adolescents’ PA was at a very low level. Higher levels of PA were associated with better mood states in children and adolescents. There were also differences in PA and mood states between children and adolescents of different sexes and different grades in primary schools. Boys and students in Grade 4 of primary school had less PA and higher levels of mood disturbances. At present, children and adolescents should participate more PA and stay in good mood states during the social isolation process of the COVID-19 epidemic to avoid harm to their health. In the future, in response to similar emergency public health situations, relevant departments should also pay attention to the impact of policy implementation on PA and mood health.

## Figures and Tables

**Table 1:** Descriptive characteristics of study subjects (*N* = 9979).

	Age (Years) M ± SD	*n*	%
**Sex**			
Boys	11.61 ± 1.24	5131	51.42%
Girls	11.65 ± 1.22	4848	48.58%
**Age (years)**			
9 years	/	435	4.36%
10 years	/	1559	15.62%
11 years	/	2334	23.39%
12 years	/	3108	31.15%
13 years	/	2036	20.40%
14 years	/	507	5.08%
**Grade**			
Grade 4	10.59 ± 0.94	3764	37.72%
Grade 5	11.77 ± 0.82	3126	31.33%
Grade 6	12.74 ± 0.77	3089	30.95%
**Wake-up time**			
Before 6:00	/	96	0.96%
6:00–8:00	/	7191	72.06%
8:00–10:00	/	2606	26.12%
After 10:00	/	86	0.86%
**Bedtime**			
20:00–21:00	/	2815	28.21%
21:00–22:00	/	5364	53.75
22:00–23:00	/	1688	16.92%
After 23:00	/	112	1.12%

Notes: M ± SD = Mean ± Standard deviation.

**Table 2:** Comparison of physical activity and mood states between different sexes (*N* = 9979).

	Total (*n* = 9979)	Boys (*n* = 5131)	Girls (*n* = 4848)
**Physical Activity** ^a^ **(** MET *-min/week)			
Moderate and Vigorous	1193.02 ± 1621.88	1186.62 ± 1670.81	1199.79 ± 1568.58 **
Vigorous	510.40 ± 934.18	526.95 ± 978.80	492.88 ± 884.29
Moderate	394.03 ± 674.68	375.70 ± 687.06	413.42 ± 660.84 **
Walking	288.60 ± 613.08	283.98 ± 610.59	293.49 ± 615.73
**Profile of Mood States**			
Tension	2.61 ± 2.50	2.64 ± 2.50	2.59 ± 2.50
Anger	2.21 ± 2.42	2.28 ± 2.46	2.14 ± 2.37 **
Fatigue	1.99 ± 2.23	2.05 ± 2.27	1.90 ± 2.20 **
Depression	1.83 ± 2.25	1.88 ± 2.25	1.78 ± 2.23 **
Confusion	3.94 ± 1.90	3.98 ± 1.94	3.84 ± 1.86 **
Vigor	4.03 ± 2.09	4.02 ± 2.10	4.04 ± 2.07
Self-esteem	4.24 ± 1.95	4.22 ± 1.96	4.25 ± 1.93
Total mood disturbance	104.28 ± 10.22	104.58 ± 10.23	103.95 ± 10.19 **

Notes: *: Metabolic Equivalent Task; **: *p* < 0.01; ^a^: according to the official IPAQ–SF scoring protocol, the PA levels are divided into high, moderate, and low three PA groups. The numbers and percentage of participants in each group have been indicated.

**Table 3:** The relationship between physical activity and mood states in different Grade groups (*N* = 9979) (x ± s).

	Grade 4 (*n* = 3764)	Grade 5 (*n* = 3126)	Grade 6 (*n* = 3089)
**Physical Activity** **(MET-min/week)**			
Moderate and vigorous	1110.20 ± 1563.40 **	1212.65 ± 641.91	1274.06 ± 666.64 ^@@^
Vigorous	479.02 ± 900.50	522.47 ± 968.54	536.41 ± 938.29 ^@@^
Moderate	359.73 ± 640.25 **	396.18 ± 663.36	433.63 ± 723.16 ^@@^
Walking	271.45 ± 595.54	294.00 ± 615.52	304.02 ± 631.09 ^@@^
**Profile of Mood States**			
Tension	2.62 ± 2.50	2.58 ± 50	2.64 ± 2.49
Anger	2.30 ± 2.44 **	2.10 ± 2.36	2.22 ± 2.45
Fatigue	2.62 ± 2.50	2.58 ± 2.50	2.64 ± 2.49 ^##^
Depression	1.89 ± 2.26 **	1.72 ± 2.18	1.86 ± 2.30 ^#^
Confusion	4.03 ± 1.96 **	3.87 ± 1.87 ^@@^	3.82 ± 1.86
Vigor	3.00 ± 2.06	4.07 ± 2.09	4.03 ± 2.13 ^##^
Self-esteem	4.18 ± 1.91 **	4.35 ± 1.97	4.20 ± 1.97
Total Mood Disturbance	104.63 ± 10.26 **	103.77 ± 10.03	104.37 ± 10.33

Notes: **: very statistically significant difference between Grade 4 and Grade 5 (*p* < 0.01); ^@@^: very statistically significant difference between Grade 4 and Grade 6 (*p* < 0.01); ^#^: statistically significant difference between Grade 5 and Grade 6 (*p* < 0.05); ^##^: very statistically significant difference between Grade 5 and Grade 6 (*p* < 0.01).

**Table 4:** The relationship between physical activity levels and mood states.

	Low (*n* = 5458)	Moderate (*n* = 2574)			High (*n* = 1947)		
		B	SE	*p*	B	SE	*p*
Tension	Ref.	−0.178	0.145	0.222	0.106	0.162	0.513
Anger	Ref.	−0.603	0.140	0.002 **	−0.149	0.157	0.343
Fatigue	Ref.	0.145	0.130	<0.001 **	−0.127	0.145	<0.001 **
Depression	Ref.	−0.556	0.130	<0.001 **	−0.112	0.145	<0.001 **
Confusion	Ref.	0.073	0.111	0.015 *	0.283	0.124	<0.001 **
Vigor	Ref.	0.693	0.120	<0.001 **	0.679	0.134	<0.001 **
Self-esteem	Ref.	0.615	0.112	<0.001**	0.630	0.125	<0.001 **
Total Mood Disturbance	Ref.	−3.031	0.590	<0.001**	−1.309	0.659	0.047 *

Notes: * *p* < 0.05, ** *p* < 0.01; B = regression coefficient and intercept; SE = standard error; *p* = *p*-value.

## References

[1] Khachfe Hussein H., Mohamad C., Julie S., Hamza S., Eldeen M.B., Fares M. (2020). An epidemiological study on COVID-19: A rapidly spreading disease. Cureus.

[2] Wang C., Horby P.W., Hayden F.G., Gao G.F. (2020). A novel coronavirus outbreak of global health concern. Lancet.

[3] Castañeda-Babarro A., Arbillaga-Etxarri A., Gutiérrez-Santamaría B., Coca A. (2020). Physical activity change during COVID-19 confinement. Int. J. Environ. Res. Public Health.

[4] Clay J.M., Parker M.O. (2020). Alcohol use and misuse during the COVID-19 pandemic: A potential public health crisis?. Lancet Public Health.

[5] Yanovski J.A., Yanovski S.Z., Sovik K.N., Nguyen T.T., O’Neil P.M., Sebring N.G. (2000). A prospective study of holiday weight gain. N. Engl. J. Med..

[6] Who Mental Health (2020). Mental Health and Psychosocial Considerations during the COVID-19 Outbreak. https://www.who.int/docs/default-source/coronaviruse/mental-health-considerations.pdf.

[7] Altena E., Baglioni C., Espie C.A., Ellis J., Gavriloff D., Holzinger B., Schlarb A., Frase L., Jernelöv S., Riemann D. (2020). Dealing with sleep problems during home confinement due to the COVID-19 outbreak: Practical recommendations from a task force of the European CBT-I Academy. J. Sleep Res..

[8] Jiménez-Pavón D., Carbonell-Baeza A., Lavie C.J. (2020). Physical exercise as therapy to fight against themental and physical consequences of COVID-19 quarantine: Special focus in older people. Prog. Cardiovasc. Dis..

[9] Steinacker J.M., Bloch W., Halle M., Mayer F., Meyer T., Hirschmüller A., Roecker K., Wolfarth B., Nieß A., Reinsberger C. (2020). Merkblatt: Gesundheitssituation für Sportler durch die aktuelle Coronavirus- Pandemie (SARS-CoV-2/COVID-19). Dtsch. Z. Sport..

[10] Bloch W., Halle M., Steinacker J.M. (2020). Sport in times of Corona <Sport in Zeiten von Corona>. Ger. J. Sports Med..

[11] World Health Organization (2012). Global Recommendations on Physical Activity for Health. http://www.mydialogue.info/files/.

[12] Shen H., Yan J., Hong J.-T., Clark C., Yang X.-N., Liu Y., Chen S.-T. (2020). Prevalence of Physical Activity and Sedentary Behavior among Chinese Children and Adolescents: Variations, Gaps, and Recommendations. Int. J. Environ. Res. Public Health.

[13] Hallal P.C., Andersen L.B., Bull F.C., Guthold R., Haskell W., Ekelund U. (2012). Global Physical activity levels: Surveillance progress, pitfalls, and propescts. Lancet.

[14] Omorou A.Y., Langlois J., Lecomte E., Vuillemin A., Briançon S. (2015). Adolescents’ Physical Activity and Sedentary Behavior: A Pathway in Reducing Overweight and Obesity: The PRALIMAP 2-Year Cluster Randomized Controlled Trial. J. Phys. Act. Health.

[15] Pretty J., Peacock J., Sellens M., Griffin M. (2005). The mental and physical health outcomes of green exercise. Int. J. Environ. Health Res..

[16] Wadolowska L., Kowalkowska J., Lonnie M., Czarnocinska J., Jezewska-Zychowicz M., Babicz-Zielinska E. (2016). Associations between physical activity patterns and dietary patterns in a representative sample of Polish girls aged 13–21 years: A cross-sectional study (GEBaHealth Project). BMC Public Health.

[17] Brooks S.K., Webster R.K., Smith L.E., Woodland L., Wessely S., Greenberg N., Rubin G.J. (2020). The psychological impact of quarantine and how to reduce it: Rapid review of the evidence. Lancet.

[18] McAlonan G.M., Murphy D.G., Edwards A.D. (2020). Multidisciplinary research priorities for the COVID-19 pandemic. Lancet Psychiatry.

[19] Kang L., Ma S., Chen M., Yang J., Wang Y., Li R., Yao L., Bai H., Cai Z., Xiang Y.B. (2020). Impact on mental health and perceptions of psychological care among medical and nursing staff in Wuhan during the 2019 novel coronavirus disease outbreak: A cross-sectional study. Brain Behav. Immun..

[20] Wang C., Pan R., Wan X., Tan Y., Xu L., McIntyre R.S., Choo F.N., Tran B., Ho R., Sharma V.K. (2020). A longitudinal study on the mental health of general population during the COVID-19 epidemic in China. Brain Behav. Immun..

[21] Qiu J., Shen B., Zhao M., Wang Z., Xie B., Xu Y. (2020). A nationwide survey of psychological distress among Chinese people in the COVID-19 epidemic: Implications and policy recommendations. Gen. Psychiatry.

[22] Wang C., Pan R., Wan X., Tan Y., Xu L., Ho C.S., Ho R.C. (2020). Immediate Psychological Responses and Associated Factors during the Initial Stage of the 2019 Coronavirus Disease (COVID-19) Epidemic among the General Population in China. Int. J. Environ. Res. Public Health.

[23] Peluso M.A.M., de Andrade L.H.S.G. (2005). Physical activity and mental health: The association between exercise and mood. Clinics.

[24] Jiang B., Zhu B. (1997). Mental health of university students in Shanghai and its relationship with physical exercise. Psychol. Sci..

[25] Norris R., Carroll D., Cochrane R. (1992). The effects of physical activity and exercise training on psychological stress and well-being in an adolescent population. J. Psychosom. Res..

[26] Rodriguez-Ayllon M., Cadenas-Sánchez C., Estévez-López F., Muñoz N.E., Mora-Gonzalez J., Migueles J.H., Molina-García P., Henriksson H., Mena-Molina A., Martínez-Vizcaíno V. (2019). Role of Physical Activity and Sedentary Behavior in the Mental Health of Preschoolers, Children and Adolescents: A Systematic Review and Meta-Analysis. Sports Med..

[27] Craig C.L., Marshall A.L., Sjöström M., Bauman A.E., Booth M.L., Ainsworth B.E., Pratt M., Ekelund U., Yngve A., Sallis J.F. (2003). International Physical Activity Questionnaire: 12-Country Reliability and Validity. Med. Sci. Sports Exerc..

[28] Fernández-Bustos J.G., Infantes-Paniagua Á., Gonzalez-Martí I., Contreras-Jordán O.R. (2019). Body Dissatisfaction in Adolescents: Differences by Sex, BMI and Type and Organisation of Physical Activity. Int. J. Environ. Res. Public Health.

[29] Srivastav A.K., Sharma N., Samuel A.J. Impact of Coronavirus Disease-19 (COVID-19) Lockdown on Physical Activity and Energy Expenditure Among Physiotherapy Professionals and Students Using Web-Based open E-Survey Sent through WhatsApp, Facebook and Instagram Messengers. https://pubmed.ncbi.nlm.nih.gov/32838062/.

[30] Nyenhuis D.L., Yamamoto C., Luchetta T., Terrien A., Parmentier A. (1999). Adult and geriatric normative data and validation of the profile of mood states. J. Clin. Psychol..

[31] McNair D.M., Lorr M., Droppleman L.F. (1971). Manual for the Profile of Mood States.

[32] DeMello M.M., Pinto B.M., Dunsiger S.I., Shook R.P., Burgess S., Hand G.A., Blair S.N. (2018). Reciprocal relationship between sedentary behavior and mood in young adults over one-year duration. Ment. Health Phys. Act..

[33] Lippi G., Henry B.M., Sanchis-Gomar F. (2020). Physical inactivity and cardiovascular disease at the time of coronavirus disease 2019 (COVID-19). Eur. J. Prev. Cardiol..

[34] Schrempft S., Jackowska M., Hamer M., Steptoe A. (2019). Associations between social isolation, loneliness, and objective physical activity in older men and women. BMC Public Health.

[35] Kwon S., Andersen L.B., Grøntved A., Kolle E., Cardon G., Davey R., Kriemler S., Northstone K., Page A.S., Puder J.J. (2019). A closer look at the relationship among accelerometer-based physical activity metrics: ICAD pooled data. Int. J. Behav. Nutr. Phys. Act..

[36] Zhang Z.H., Li H.J., Slapsinskaite A., Zhang T., Zhang L., Gui C.Y. (2020). Accelerometer-measured physical activity and sedentary behavior in Chinese children and adolescents: A systematic review and meta-analysis. Public Health.

[37] López-Sánchez G.F., Pardhan S., Trott M., Sánchez-Castillo S., Jackson S.E., Tully M., Gorely T., López-Bueno R., Veronese N., Skalska M. (2020). The Association Between Physical Activity and Cataracts Among 17,777 People Aged 15-69 Years Residing in Spain. Ophthalmic Epidemiol..

[38] Maugeri G., Castrogiovanni P., Battaglia G., Pippi R., D’Agata V., Palma A., Di Rosa M., Musumeci G. (2020). The impact of physical activity on psychological health during Covid-19 pandemic in Italy. Heliyon.

[39] Di Renzo L., Gualtieri P., Pivari F., Soldati L., Attinà A., Cinelli G., Leggeri C., Caparello G., Barrea L., Scerbo F. (2020). Eating habits and lifestyle changes during COVID-19 lockdown: An Italian survey. J. Transl. Med..

[40] Gallo L.A., Gallo T.F., Young S.L., Moritz K.M., Akison L.K. (2020). The Impact of Isolation Measures Due to COVID-19 on Energy Intake and Physical Activity Levels in Australian University Students. Nutrients.

[41] Lesser I.A., Nienhuis C.P. (2020). The Impact of COVID-19 on Physical Activity Behavior and Well-Being of Canadians. Int. J. Environ. Res. Public Health.

[42] Molanorouzi K., Khoo S., Morris T. (2015). Motives for adult participation in physical activity: Type of activity, age, and gender. BMC Public Health.

[43] Lustyk M.K.B., Widman L., Paschane A.A., Olson K.C. (2004). Physical activity and quality of life: Assessing the influence of activity frequency, intensity, volume, and motives. Behav. Med..

[44] Markland D., Hardy L. (1993). The exercise motivations inventory: Preliminary development and validity of a measure of individuals’ reasons for participation in regular physical exercise. Personal. Individ. Differ..

[45] Huang W.Y., Wong S.H., Salmon J. (2013). Correlates of physical activity and screen-based behaviors in Chinese children. J. Sci. Med. Sport.

[46] Li W., Procter-Gray E., Churchill L., Crouter S.E., Kane K., Tian J., Franklin P.D., Ockene J.K., Gurwitz J. (2017). Gender and Age Differences in Levels, Types and Locations of Physical Activity among Older Adults Living in Car-Dependent Neighborhoods. J. Frailty Aging.

[47] Peral-Suárez Á., Cuadrado-Soto E., Perea J.M., Navia B., López-Sobaler A.M., Ortega R. (2020). Physical activity practice and sports preferences in a group of Spanish schoolchildren depending on sex and parental care: A gender perspective. BMC Pediatr..

[48] Groth S.W., Rhee H., Kitzman H. (2016). Relationships among obesity, physical activity and sedentary behavior in young adolescents with and without lifetime asthma. J. Asthma.

[49] Guddal M.H., Stensland S.Ø., Småstuen M.C., Johnsen M.B., Heuch I., Zwart J.A., Storheim K. (2020). Obesity in Young Adulthood: The Role of Physical Activity Level, Musculoskeletal Pain, and Psychological Distress in Adolescence (The HUNT-Study). Int. J. Environ. Res. Public Health.

[50] Königstein K., Infanger D., Klenk C., Carrard J., Hinrichs T., Schmidt-Trucksäss A. (2020). Physical activity is favorably associated with arterial stiffness in patients with obesity and elevated metabolic risk. Int. J. Clin. Pract..

[51] Uddin R., Burton N.W., Maple M., Khan S.R., Tremblay M.S., Khan A. (2020). Low physical activity and high sedentary behaviour are associated with adolescents’ suicidal vulnerability: Evidence from 52 low- and middle-income countries. Acta Paediatr..

[52] Lau E.Y., Dowda M., McIver K.L., Pate R.R. (2017). Changes in Physical Activity in the School, Afterschool, and Evening Periods during the Transition from Elementary to Middle School. J. Sch. Health.

[53] Mooses K., Mägi K., Riso E.M., Kalma M., Kaasik P., Kull M. (2017). Objectively measured sedentary behaviour and moderate and vigorous physical activity in different school subjects: A cross-sectional study. BMC Public Health.

[54] Sawa S., Hashizume K., Abe T., Kusaka Y., Fukazawa Y., Hiraku Y., Hagihara A. (2019). Pathway linking physical activity, sleep duration, and breakfast consumption with the physical/psychosocial health of schoolchildren. J. Child Health. Care.

[55] Zhang Y., Zhang H., Ma X., Di Q. (2020). Mental Health Problems during the COVID-19 Pandemics and the Mitigation Effects of Exercise: A Longitudinal Study of College Students in China. Int. J. Environ. Res. Public Health.

[56] Bäuerle A., Teufel M., Musche V., Weismüller B., Kohler H., Hetkamp M., Dörrie N., Schweda A., Skoda E.-M. (2020). Increased generalized anxiety, depression and distress during the COVID-19 pandemic: A cross-sectional study in Germany. J. Public Health.

[57] Liu N., Zhang F., Wei C., Jia Y., Shang Z., Sun L., Wu L., Sun Z., Zhou Y., Wang Y. (2020). Prevalence and predictors of PTSS during COVID-19 outbreak in China hardest-hit areas: Gender differences matter. Psychiatry Res..

[58] Lim G.Y., Tam W.W., Lu Y., Ho C.S., Zhang M.W., Ho R.C. (2018). Prevalence of depression in the community from 30 countries between 1994 and 2014. Sci. Rep..

[59] Huang Y., Zhao N. (2020). Generalized anxiety disorder, depressive symptoms and sleep quality during COVID-19 outbreak in China: A web-based cross-sectional survey. Psychiatry Res..

[60] Ussher M.H., Owen C.G., Cook D.G., Whincup P.H. (2007). The relationship between physical activity, sedentary behaviour and psychological wellbeing among adolescents. Soc. Psychiatry Psychiatr. Epidemiol..

[61] Calisher C., Carroll D., Colwell R., Corley R.B., Daszak P., Drosten C., Enjuanes L., Farrar J., Field H., Golding J. (2020). Statement in support of the scientists, public health professionals, and medical professionals of China combatting COVID-19. Lancet.

[62] Ahn S., Fedewa A. (2011). A meta-analysis of the relationship between children’s physical activity and mental health. J. Pediatr. Psychol..

[63] Spruit A., Assink M., van Vugt E., van der Put C., Stams G.J. (2016). The effects of physical activity interventions on psychosocial outcomes in adolescents: A meta-analytic review. Clin. Psychol. Rev..

[64] Brown H.E., Pearson N., Braithwaite R.E., Brown W.J., Biddle S.J. (2013). Physical activity interventions and depression in children and adolescents. Sports Med..

[65] Babic M.J., Morgan P.J., Plotnikoff R.C., Lonsdale C., White R.L., Lubans D.R. (2014). Physical activity and physical self-concept in youth: Systematic review and meta-analysis. Sports Med..

[66] Liu M., Wu L., Ming Q. (2015). How Does Physical Activity Intervention Improve Self-Esteem and Self-Concept in Children and Adolescents? Evidence from a Meta-Analysis. PLoS ONE.

[67] Ruotsalainen H., Kyngäs H., Tammelin T., Kääriäinen M. (2015). Systematic review of physical activity and exercise interventions on body mass indices, subsequent physical activity and psychological symptoms in overweight and obese adolescents. J. Adv. Nurs..

[68] Poitras V.J., Gray C.E., Borghese M.M., Carson V., Chaput J.P., Janssen I., Katzmarzyk P.T., Pate R.R., Gorber S.C., Kho M.E. (2016). Systematic review of the relationships between objectively measured physical activity and health indicators in school-aged children and youth. Appl. Physiol. Nutr. Metab..

[69] Lubans D., Richards J., Hillman C., Faulkner G., Beauchamp M., Nilsson M., Kelly P., Smith J., Raine L., Biddle S. (2016). Physical Activity for Cognitive and Mental Health in Youth: A Systematic Review of Mechanisms. Pediatrics.

[70] Wagnsson S., Lindwall M., Gustafsson H. (2014). Participation in organized sport and self-esteem across adolescence: The mediating role of perceived sport competence. J. Sport Exerc. Psychol..

[71] Reigal R.E., Videra A., Gil J. (2014). Physical exercise, general self-efficacy and life satisfaction in adolescence. Rev. Int. Med. y Ciencias la Act. Fis. y del Deport..

[72] Ho F.K., Louie L.H., Chow C.B., Wong W.H., Ip P. (2015). Physical activity improves mental health through resilience in Hong Kong Chinese adolescents. BMC Pediatr..

